# Nutrition, Health, and Disease: Role of Selected Marine and Vegetal Nutraceuticals

**DOI:** 10.3390/nu12030747

**Published:** 2020-03-11

**Authors:** Lola Corzo, Lucía Fernández-Novoa, Iván Carrera, Olaia Martínez, Susana Rodríguez, Ramón Alejo, Ramón Cacabelos

**Affiliations:** 1 EuroEspes Biomedical Research Center, Institute of Medical Science and Genomic Medicine, 15165 Bergondo, Corunna, Spain; biotecnologiasalud@euroespes.com (I.C.); epigenetica@euroespes.com (O.M.); analisis2@euroespes.com (S.R.); rcacabelos@euroespes.com (R.C.); 2 EuroEspes Biotechnology, Polígono Industrial de Bergondo, Parroquia de Guísamo, A-6, 15165 Bergondo, Corunna, Spain; genetica@ebiotec.com (L.F.-N.); direcciontecnica@ebiotec.com (R.A.)

**Keywords:** bioproduct, cancer, cardiovascular disease, immune system, immunomodulation, menopause, nutraceutical, osteoporosis, Parkinson disease, prevention

## Abstract

The investigation of new alternatives for disease prevention through the application of findings from dietary and food biotechnology is an ongoing challenge for the scientific community. New nutritional trends and the need to meet social and health demands have inspired the concept of functional foods and nutraceuticals which, in addition to their overall nutritional value, present certain properties for the maintenance of health. However, these effects are not universal. Nutrigenetics describes how the genetic profile has an impact on the response of the body to bioactive food components by influencing their absorption, metabolism, and site of action. The EbioSea Program, for biomarine prospection, and the Blue Butterfly Program, for the screening of vegetable-derived bioproducts, have identified a new series of nutraceuticals, devoid of side effects at conventional doses, with genotype-dependent preventive and therapeutic activity. Nutrigenomics and nutrigenetics provide the opportunity to explore the inter-individual differences in the metabolism of and response to nutrients, achieving optimal results. This fact leads to the concept of personalized nutrition as opposed to public health nutrition. Consequently, the development and prescription of nutraceuticals according to the individual genetic profile is essential to improve their effectiveness in the prevention and natural treatment of prevalent diseases.

## 1. Introduction

According to the World Health Organization (WHO), ‘Health is a state of complete physical, mental and social well-being and not merely the absence of disease or infirmity’ [1]. Lifestyle behavior can be beneficial for health, but can also damage it. Specifically, good nutrition is a fundamental element of good health. Poor nutrition including undernutrition and overnutrition can reduce immunity, increase vulnerability to preventable diseases, alter physical and mental development, and impact future generations. Rapid changes in diet and lifestyle that have occurred due to industrialization, urbanization, economic development, and market globalization have arisen over the past decade, causing a significant impact on the health and nutritional status of populations. Due to these changes in dietary and lifestyle patterns, chronic diseases or conditions (including obesity, diabetes mellitus, cardiovascular disease (CVD), hypertension and stroke, and some types of cancer) are becoming increasingly significant causes of disability and premature death in both developing and newly developed countries, placing additional burdens on already overtaxed national health budgets [2]. Scientific evidence increasingly supports the idea that diet has a major influence on health throughout life, and that diet is an important modifiable determinant for disease prevention. Eating habits not only affect current health, but may determine the development of future diseases in later stages of life [3,4]. Many of the pathological mechanisms involved in disease development may be modified by specific bioactive compounds of food that could change health status including disease prevention and progression. The appearance of genomics has presented an opportunity to explore the inter-individual differences in the metabolism of and response to nutrients. This has led to the concept of personalized nutrition as opposed to public health “one-size-fits-all” nutrition. It has been accepted that personalized diets aimed at individuals and subgroups, in order to prevent chronic diseases, is a necessary approach to prevention. Genetic variants that influence nutrient metabolism have been identified, and individual variants have been linked to the risk of pathologies such as cancer and cardiovascular disease [5].

## 2. Nutraceuticals, Food Supplements, and Functional Foods

The investigation of novel alternatives for disease prevention through the application of findings from food biotechnology is an ongoing challenge for the scientific community. New nutritional trends and the need to meet social and health requirements have promoted the concept of functional foods and nutraceuticals, which, in addition to their overall nutritional value, present certain properties for the maintenance of health. 

There are widespread inconsistencies and contradictions in the definitions of nutraceuticals and functional foods. The term nutraceutical was coined from “nutrition” and “pharmaceutical” in 1989 by Dr. Stephen DeFelice, President of the Foundation for Innovation in Medicine (FIM), in Cranford, New Jersey, USA. Dr. DeFelice defined nutraceuticals as “a food or part of a food that provides medical or health benefits, including disease prevention and/or treatment” [6]. DeFelice defined a concept that has been used since the Paleolithic. Hippocrates (400 B.C.) said: “Let food be your medicine and medicine your food”. Nearly 2500 years after Hippocrates, we remain mired in conceptual confusion. 

“Nutraceutical” is defined in the Oxford English Dictionary as “a foodstuff, food additive, or dietary supplement that has beneficial physiological effects but is not essential to the diet”. In the proposed U.S. Nutraceutical Research and Education Act (106^th^ Congress of 1999–2000), it was defined as “a dietary supplement, food or medical food that has a benefit, which prevents or reduces the risk of a disease or health condition, including the management of a disease or health condition or the improvement of health; and is safe for human consumption in the quantity, and with the frequency required to realize such properties” [7]. The European Nutraceutical Association defines nutraceuticals as “nutritional products which have effects that are relevant to health, which are not synthetic substances or chemical compounds formulated for specific indications, containing nutrients (partly in concentrated form)”. Health Canada has defined a functional food as one that “is similar in appearance to, or may be, a conventional food that is consumed as part of a usual diet, and is demonstrated to have physiological benefits and/or reduce the risk of chronic disease beyond basic nutritional functions” [8]. An accompanying definition says that a nutraceutical is “a product isolated or purified from foods that is generally sold in medicinal forms not usually associated with food, demonstrated to have a physiological benefit or provide protection against chronic disease”. This seems to differentiate the two terms “functional food” and “nutraceutical”. 

Nutraceuticals are biological substances extracted from natural sources by non-denaturing processes to preserve their original properties without any chemical manipulation. The extracts are studied in animals and humans in order to discover their biological properties, as is done with drugs. Once their properties have been documented, they are marketed to be consumed by humans as part of the diet. Nutraceuticals are usually present as concentrated extracts which, taken at a dose higher than that present in the original foods, have a favorable effect on health, greater than the natural food itself could have. These are attractive products due to their natural origin, good bioavailability, and can usually be taken in the long term without any risk of side effects. 

Nutraceuticals are causing a heated debate because their concept redefines the traditional dividing line between food and medicine. The main difference is based on their origin, this being synthetic for drugs and natural for nutraceuticals. Nutraceuticals occupy the void that exists between food and drugs, and strongly claim their own legal space, taking into account their characteristics and idiosyncrasies and enabling the development of their full therapeutic potential [9,10].

## 3. The Ebiosea Program: Marine and Vegetal Nutraceutical Research

The EbioSea program was launched in the 2000s with the aim of designing a marine prospection program in the North Atlantic Ocean in search of nutraceuticals for the prevention and treatment of diseases prevalent in Western countries. Different fish and mollusk species were investigated using non-denaturing biotechnological procedures and screening protocols [11,12], and a novel category of nutraceutical compounds was characterized and represented by the lipofishins (LFs), a series of complex lipoproteins derived from marine resources [13]. E-JUR-94013 (DefenVid), E-CAB-94011 (CabyMar), E-Congerine-10423 (AntiGan), E-SAR-94010 (LipoEsar), and E-MHK-0103 (MineraXin) are the most representative LFs obtained from biomarine sources. Favalins are another novel category of nutraceuticals extracted from the structural components of the *Vicia faba* plant. E-PodoFavalin-15999 (Atremorine) is the leading compound in this series, with powerful catecholaminergic properties and neuroprotective activity on dopaminergic neurons. Basic and clinical studies with some of these products under different clinical conditions (cardiovascular disorders, dyslipidemia, cancer, Alzheimer’s disease, Parkinson’s disease, menopause) have revealed significant beneficial results. In this program, we have identified a new series of nutraceuticals, devoid of side effects at conventional doses, with preventive and/or therapeutic effects, depending upon the genetic profile of each patient [14,15,16].

### 3.1. Marine Biotechnology-Related Products

#### 3.1.1. LipoEsar (E-SAR-94010)

LipoEsar is a nutraceutical belonging to the lipofishin family that was developed by our research team in the late 1990s, with the aim of exploiting the biological properties of blue (oily) fish in order to develop a nutraceutical that maintains all the healthy properties of the original species [13]. The E-SAR-94010 extract is the structural base of LipoEsar, obtained by non-denaturing biotechnological procedures from the dorsal muscle of the species *Sardina pilchardus* Walbaum, 1792. This species is obtained in the waters of the North Atlantic Ocean, over 50 miles offshore. The nutritional composition of LipoEsar reveals a high protein content, about 65%; a high unsaturated fatty acid content, approximately 11%; and a saturated fatty acid content of 9%. The most common monounsaturated fatty acids in LipoEsar are oleic and palmitoleic acids; polyunsaturated fatty acids are represented mostly by eicosapentaenoic acid (EPA) and docosahexaenoic acid (DHA), and the most abundant saturated fatty acid is palmitic acid. LipoEsar is also rich in minerals such as phosphorous, and vitamins, principally vitamins D and B [13,16] (Table 1). In in vivo and in vitro preclinical studies, LipoEsar has been shown to be effective in: (i) reducing serum cholesterol levels; (ii) reducing serum triglyceride levels; (iii) reducing serum glucose levels; (iv) modulating different immunological parameters, increasing white blood cell, lymphocyte, and monocyte counts; and (v) reducing the number of ameboid microglial cells and modulating the release of several neuroimmunological soluble factors in the in vitro microglial cell cultures [13,17,18] (Table 2).

**Figure 1:**
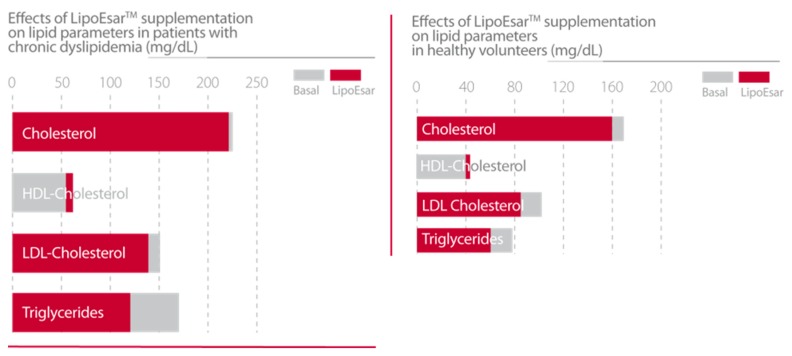
Effects of LipoEsar supplementation on lipid parameters in patients with chronic dyslipidemia (419 patients with 750 mg/day LipoEsar for three months) [19] and healthy volunteers (500 mg/day LipoEsar for two weeks) [13].

**Figure 2:**
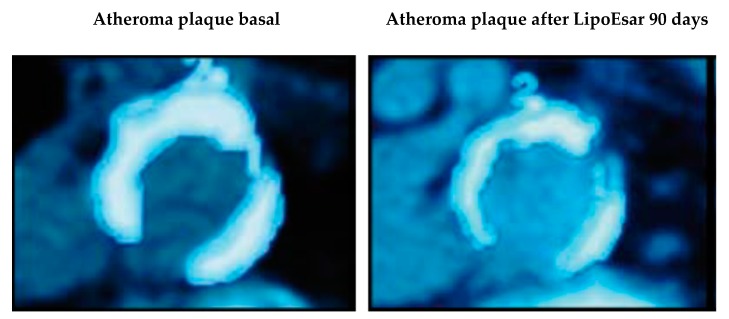
Effects of LipoEsar on the atheroma plaque of the abdominal aorta of 30 patients suffering from chronic dyslipidemia supplemented with 1500 mg/day LipoEsar for three months. Adapted from [20].

In clinical studies, LipoEsar displayed a powerful effect in reducing the impact of cardiovascular risk factors in healthy subjects and in patients with chronic hyperlipidemia (Figure 1) such as elevated levels of total cholesterol and low-density lipoprotein (LDL) cholesterol, low high-density lipoprotein (HDL) cholesterol, and the presence of high blood glucose and uric acid levels. LipoEsar also has a hepatoprotective effect, reflected by the reduction in alanine transaminase (ALT), aspartate transaminase (AST) and gamma-glutamyl transpeptidase (GGT) enzyme activities. Other relevant effects of LipoEsar are the reduction in the size of xanthelasma plaques, a typical sign of the presence of high blood cholesterol levels, and the decreased size of atherosclerotic plaques on the aortic wall in patients with chronic hyperlipidemia (Figure 2). The therapeutic response to LipoEsar in chronic hyperlipidemic patients is apolipoprotein E (*APOE)* genotype-dependent. The best responders are those patients with *APOE*-3/3 > *APOE*-3/4 > *APOE*-4/4 [13,17,19,20,21]. LipoEsar is highly effective as a co-adjuvant of statin therapy in hyperlipidemic patients, enabling a reduction in statin dose, and avoiding the deleterious effects produced by statin treatment. In a group of patients with dementia with associated hyperlipidemia whose treatment was supplemented with LipoEsar, we observed an effect similar to that seen in hyperlipidemic patients [16,17,20,22] (Table 2).

In healthy subjects supplemented with LipoEsar, we observed an increase in lymphocyte subset markers CD3, CD4, CD25, CD26, CD28, and CD56, and a reduction in CD8 and HLA-DR antigen expression. These results appear to indicate a stimulating effect of LipoEsar on the immune system in healthy volunteers [23] (Table 2).

Several possible mechanisms of action can be responsible for the effects observed in these studies. Affane et al. [24] found that sardine by-product proteins, due to their richness in certain essential amino acids, have weight-loss, lipid-lowering, antioxidant, and anti-atherogenic potentials, and contribute to improving the complications associated with obesity. The broad biological effects of fish n-3 PUFAs such as EPA ad DHA also affect blood lipids and lipoproteins, arterial blood pressure, cardiac function, arterial elasticity, endothelial function, vascular reactivity, heart electrophysiology, antiplatelet activity, and immunostimulant and anti-inflammatory effects [25,26]; all of these are directly implicated in the pathogenesis of cardiovascular disorders. PUFAs can act on lipid metabolism to reduce the activity of lipid synthesis-associated enzymes such as fatty acid synthase and stearoyl-CoA desaturase-1 [27]. However, Brown et al. [28] observed that fish omega-3 fatty acids ameliorate atherosclerosis by favorably altering monocyte subsets and limiting monocyte recruitment to aortic lesions. Fish proteins and lipids are major components of LipoEsar, which has immunoregulatory properties that could support its atheroprotective effect. Although further studies must be performed to elucidate the mechanisms by which LipoEsar acts, its effects are likely due to the bioactivity of all active components.

In conclusion, LipoEsar is an effective nutraceutical compound with important therapeutic benefits covering relevant medical specialties including the cardio- and cerebrovascular systems, the central nervous system, and antiaging. LipoEsar is an excellent source of protein, peptides, omega fatty acids, antioxidants, vitamins, minerals, and other bioactive molecules that are important for the prevention of or delay in the onset of chronic diseases such as cardio- and cerebrovascular pathologies, diseases of the central nervous system and metabolic diseases, these being the leading causes of death and disability worldwide.

#### 3.1.2. DefenVid (E-JUR-94013)

DefenVid is a nutraceutical belonging to the lipofishin family that was developed by our research team in the late 90s. The E-JUR-94013 extract is the structural base of DefenVid, and is obtained from the dorsal muscle of the marine species *Trachurus trachurus* Linnaeus, 1758 (Atlantic horse mackerel). The production of DefenVid involves non-denaturing biotechnological processes that enable the preservation of all of the healthy properties of the original species. DefenVid contains a wide variety of macronutrients and micronutrients with recognized health-enhancing properties such as natural omega-3 fatty acids; vitamins, mainly A, D, and B complex; and minerals, essentially magnesium, iron, and iodine. DefenVid is a rich source of protein, this being approximately 70% of its nutritional composition. It is well known that marine proteins are of a high quality, and upon digestion, these proteins are sources of bioactive peptides with documented favorable physiological effects such as antioxidative, antihypertensive, and other cardioprotective properties [29]. DefenVid composition has an unsaturated fatty acid content of 12% and a saturated fatty acid content of 7%. The main monounsaturated fatty acid is oleic acid and, to a lesser extent, long-chain monounsaturated fatty acids (LCMUFA); the polyunsaturated fatty acids are represented mostly by EPA and DHA, and the most abundant saturated fatty acid is palmitic acid (Table 1). DefenVid displays anti-inflammatory activity and powerful immune-enhancing properties in cases of immunodeficiency, microbial infections, and/or diseases in which there is a functional compromise of the immune system.

In vitro and in vivo studies revealed that DefenVid produced a stimulation of antibody-secreting cell response, an enhancement of phagocytosis, a modification/enhancement of cytokine production, and an improvement in the host-acquired immune responses. In vitro studies with E-JUR-94013 in peripheral blood lymphocytes from healthy volunteers showed a strong immunological effect, inducing a clear immune activation as measured by the increased levels of CD25, CD8, CD38, CD19, and HLA-DR in vitro expression on lymphocytes. In addition, there was a reduction in apoptotic CD19+CD38+ double-positive lymphocytes, indicating an increase in lymphocyte efficiency and survival [23]. In vivo studies with piglets, which were supplemented with different concentrations of E-JUR-94013 extract, showed an immune modulation effect with an increased production of all immunoglobulins, mainly affecting the IgA class [30] (Table 3).

**Figure 3:**
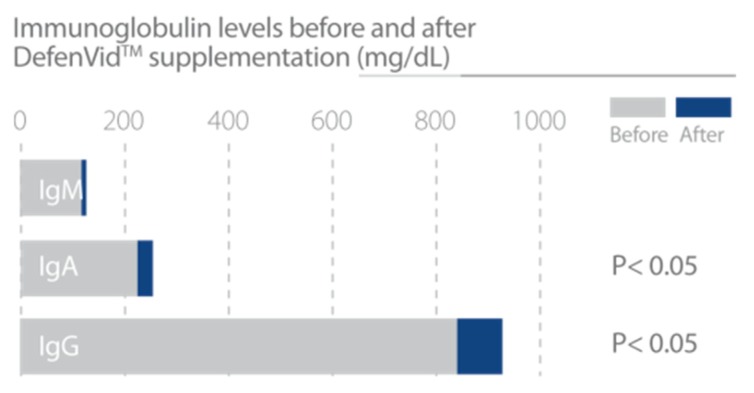
DefenVid increases immunoglobulin A, G, and M levels, enhancing humoral immune response. Study design: Two hundred and five patients with different types of white blood cell dysfunction supplemented with 750 mg/day DefenVid for three months [32].

**Figure 4:**
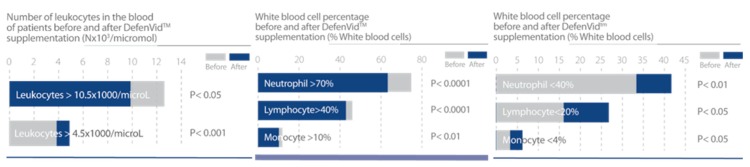
DefenVid exerts an immunomodulatory effect in patients with irregular white blood cell count. Study design: Two hundred and five patients with different types of white blood cell dysfunction supplemented with 750 mg/day DefenVid for three months [32].

In clinical studies, DefenVid was able to modulate cellular and humoral immune responses, increasing the number of all leukocyte subclasses and the serum levels of immunoglobulins A, G, and M, while displaying a slight reduction in the concentration of IgE (Figure 3). DefenVid also displayed an immunomodulatory effect, increasing the white blood cell count in immunodeficient patients and decreasing it in patients with high leukocyte counts at the baseline, returning to normal levels in both cases (Figure 4). This immunomodulatory effect is influenced, in part, by genetic polymorphisms in *IL1B*, *IL6*, and *TNF* genes, mainly involved in immune regulation and inflammation [32]. In patients with an immunodeficient phenotype, DefenVid reduces blood cholesterol levels, but this effect is influenced by genetic variants in genes associated with inflammation such as *IL1B*-T3954C, *IL6*-G174C, *IL6R*-A1510C, and *TNFA*-G308A, suggesting its usefulness in the prevention of atherogenesis [32] (Table 3).

The biological mechanisms of action of DefenVid have not been fully researched and depend on the nature of the nutrients. Immunonutrition based on proteins (arginine, RNA) and lipids (omega-3 fatty acids) was reported to reduce stress-induced immunosuppression [33]. Fish oil-enriched food has been related to a significant increase in the percent of eicosapentaenoic acid in phospholipids of white blood cells and the immune response [34], a regulatory effect of T cells as controllers of immune responses, and the modulation of Th1/Th2 differentiation and Th17 response [33]. Other authors have reported that the stimulation of lymphocyte flux and blastogenesis in the intestinal lymphatic system through the absorption of long-chain fatty acids [35] and lipoproteins are involved in the stimulation of lymphocyte function by both receptor-dependent and -independent mechanisms. The immunomodulatory effect of vitamin D is well-known, promoting dendritic cell and regulatory T-cell differentiation, and reducing T helper Th17 cell response and inflammatory cytokine secretion [36]. The immunoregulatory and anti-inflammatory activity found in DefenVid and the presence of different bioprotective substances in its composition such as PUFAs (EPA, DHA, and alpha-linolenic acid), proteins, vitamins (vitamin D and B complex), and other micronutrients may be associated therewith. Regardless of the mechanism of action, our group suggests that DefenVid might act on the systemic cellular immune system by modulating lymphocyte action and in intestinal immune function at the IgA level [32].

#### 3.1.3. CabyMar (E-CAB-94011)

CabyMar is a nutraceutical belonging to the lipofishin family that was developed by our research team in the late 1990s. CabyMar, LipoEsar, and DefenVid make up the main group of lipofishins in terms of their fatty acid content. The E-CAB-94011 extract is the structural base of CabyMar and is derived from the dorsal muscle of the marine species *Scomber scombrus* Linnaeus, 1758 (Atlantic Mackerel) with antioxidant, anti-inflammatory, and bioenergizing properties. 

CabyMar nutrient data consist of fish proteins of high biological value, essential amino acids, natural mono- and polyunsaturated fatty acids, vitamins, essentially B_12_, B_6_, D, and minerals, mostly iodine, iron, and magnesium. CabyMar is a rich source of protein, this being approximately 65% of its nutritional composition. It has an unsaturated fatty acid-content of 18% and a saturated fatty acid content of 6%. CabyMar is particularly rich in LCMUFAs, which account for 58% of total monounsaturated fatty acids, mainly represented by cis-gondoic acid (C20:1 n9) and cis-cetoleic acid (C22:1 n11). The polyunsaturated fatty acids are represented mostly by EPA and DHA (Table 1).

In human lymphocytes from healthy volunteers, E-CAB-94010 increased the expression levels of the activation markers CD25, CD8, CD38, CD19, and HLA-DR, and reduced apoptotic CD19^+^CD38^+^ double-positive lymphocytes, increasing lymphocyte efficiency and survival [23] (Table 4).

In in vivo studies with weaned farm piglets supplemented with E-CAB-94010, a health benefit and a growth promotion response were observed. The main effects of E-CAB-94010 supplementation included (i) increased mean weight and average daily gain; (ii) decreased total plasma cholesterol, LDL-cholesterol and triglycerides, and increased HDL-cholesterol; (iii) increased expression of CD4, CD8, CD25, and CD56 in peripheral blood lymphocytes; and (iv) an increase in both IgA and IgG concentrations [37] (Table 4).

**Figure 5:**
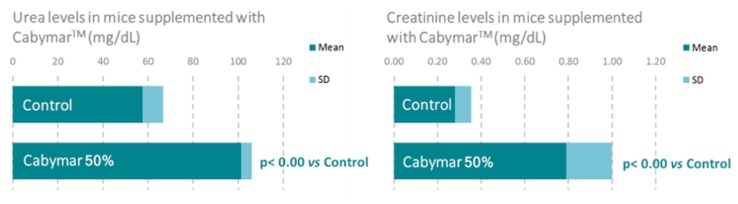
Beneficial effects of CabyMar on muscle growth in mice. Study design: 12-week randomized case-control study on a muscle development model in C57BI/6 mice supplemented with 2.5 g CabyMar in 5 g total diet/day.

**Figure 6:**
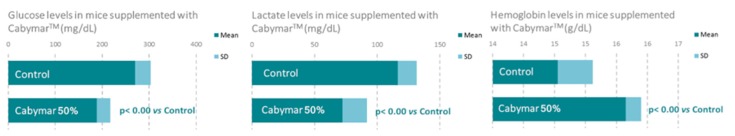
Beneficial effects of CabyMar on mitochondrial bioenergetics in mice. Study design: 12-week randomized case-control study on a muscle development model in C57BI/6 mice supplemented with 2.5 g CabyMar in 5 g total diet/day.

**Figure 7:**
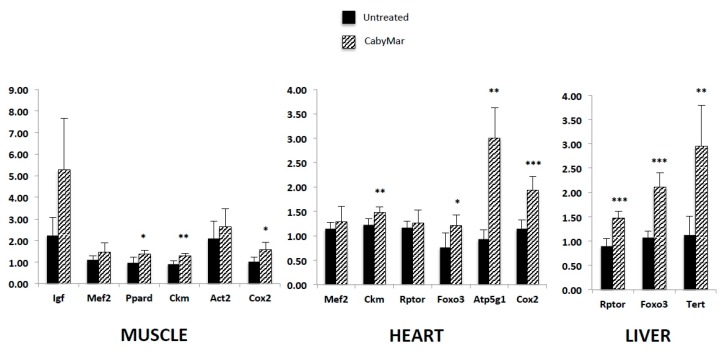
Gene expression patterns of relevant biomarkers involved in muscle growth, mitochondrial bioenergetics, and longevity in muscle, heart, and liver of CabyMar-treated mice vs. untreated animals. Study design: 12-week randomized case-control study on a muscle development model in C57BI/6 mice supplemented with 2.5 g CabyMar in 5 g total diet/day. Data correspond to mean ± STD. Statistical significance was tested with the nonparametric Mann–Whitney U test (* *p < 0.05*; ** *p < 0.01*; *** *p < 0.001*). *Atp5g1*, ATP synthase, H+ transporting, mitochondrial Fo complex subunit C1 (subunit 9); *Ckm*, creatine kinase, M-type; *Cox2*, cytochrome c oxidase, subunit II; *Foxo3*, forkhead box O3; *Igf*, insulin-like growth factor 1; *Mef2*, myocyte enhancer factor 2; *Mstn*, myostatin; *Ppard*, peroxisome proliferator-activated receptor delta; *Rptor*, regulatory-associated protein of MTOR complex 1; *Tert*, telomerase reverse transcriptase. Adapted from [16].

A recent study in mice incorporating biochemical, histological, and gene expression data confirmed the aforementioned metabolic benefits of CabyMar, and emphasized the biochemical markers of muscle mass growth, mitochondrial bioenergetics, and longevity (Figure 5 Figure 6 Figure 7). The gene expression study indicated increased expression of genes related to muscle growth, response to exercise, mitochondrial bioenergetics, and fatty acid metabolism in muscle and heart tissues (Figure 7) [16] (Table 4).

Rossignoli et al. [38] found similar changes in mitochondrial mechanisms induced by dietary supplementation with linoleic acid and/or EPA/DHA that may be associated with elevated body energy expenditure in mice. Dietary supplementation with conjugated linoleic acid or EPA/DHA increased body VO_2_ consumption, VCO_2_ production, and energy expenditure, with fish oil (EPA/DHA) being the most potent. Fish protein was proven to induce fast-muscle hypertrophy, and the enhancement of basal energy expenditure by muscle hypertrophy; the increase in muscle glucose uptake reduces liver lipids and serum glucose levels [39]. Recently, Zhao et al. [40] demonstrated that peptides from the muscle of the Spanish mackerel have strong antioxidant activity, which correlated with our biochemical studies with CabyMar. A study conducted with human samples from a UK biobank found that in regular meat-eaters, around 25% of their total energy was derived from meat, fish, dairy and plant milk, cheese, yogurt, and eggs [41]. An association study between protein intake and muscle mass in 31,278 men and 45,355 women from the Lifelines Cohort suggests that total protein and animal protein intake, and in particular fish/meat/egg protein intake may be important for building and preserving muscle mass [42]. Multiple lines of evidence suggest that dietary *n*-3 PUFAs provide an essential link between musculoskeletal and cardio-metabolic health in older adults [43]. Data from a clinical trial in subjects with insulin-resistant/metabolic syndrome demonstrated a beneficial effect of high-monounsaturated fatty acid (HMUFA) diet [44]. In conclusion, the high content of bioactive nutrients such as unsaturated fatty acids and proteins included in the CabyMar composition could support its energizing effect. 

#### 3.1.4. AntiGan (E-Congerine-10423)

AntiGan is a nutraceutical belonging to the lipofishin family, which was developed by means of non-denaturing biotechnological procedures from the skin and muscular structures of the species *Conger conger*. The E-Congerine-10423 extract is the structural base of AntiGan. Of particular note in the nutritional composition of E-Congerine-10423 is its high protein content (80%), its considerable unsaturated fatty acid content (70% of the total fat), the presence of vitamins A and D in large quantities, B vitamins, principally B1 and B3, and minerals such as phosphorus, potassium, and magnesium. The main monounsaturated fatty acid in AntiGan is oleic acid; the polyunsaturated fatty acids are represented mostly by EPA, DHA, and linoleic acid, and the most abundant saturated fatty acid is palmitic acid (Table 1). 

**Figure 8:**
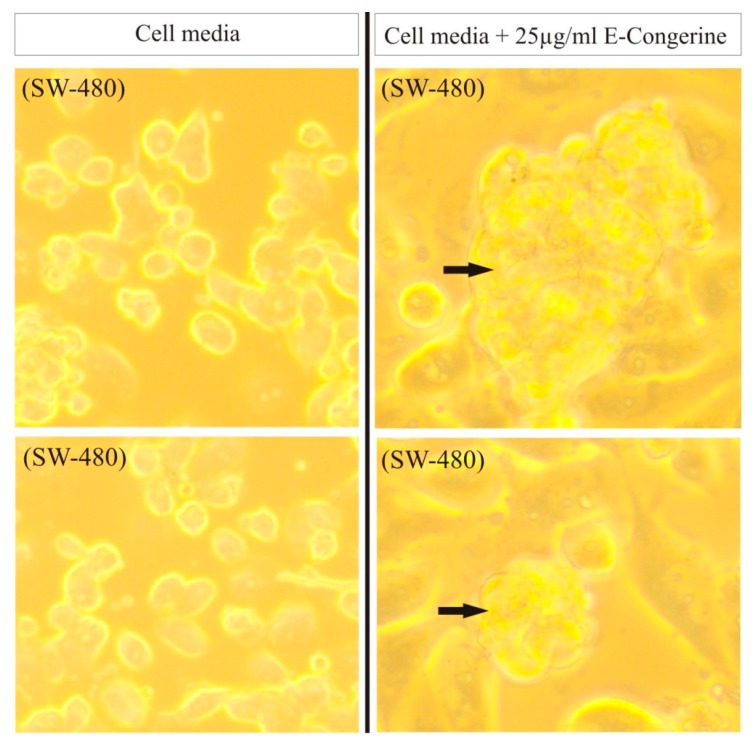
Effect of AntiGan on SW-480 cell cultures (human colon adenocarcinoma). AntiGan induced the formation of nuclear condensation, suggesting anti-tumoral activity. Adapted from [46].

**Figure 9:**
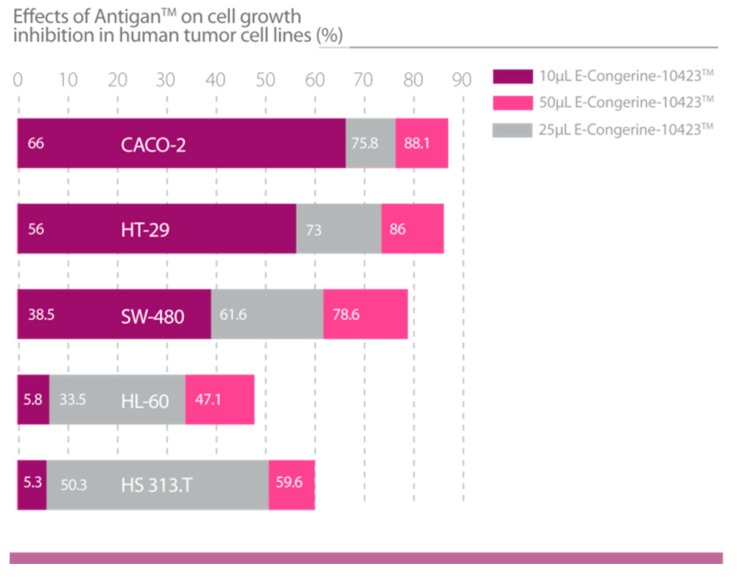
Effects of AntiGan on cell growth inhibition in human tumor cell lines (HL-60: Acute promyelocytic leukemia; HS313.T: Lymphoma; Caco-2: Colorectal adenocarcinoma; HT-29: Colorectal adenocarcinoma; and SW-480: Colorectal adenocarcinoma) [46].

The extract displays cytotoxic and apoptotic activity in different human tumor cell lines (Figure 8), with a significant effect against colorectal adenocarcinoma, sarcoma, and liposarcoma (Figure 9) [29,30,45,46] (Table 5). 

In an animal model of colonic inflammation, inducing colitis-associated dysplasia and/or tumor hallmarks by exposure to 2% dextran sodium sulfate (DSS), AntiGan, included in the diet of mice, decreased the number and severity of histopathological lesions caused by exposure to DSS [32,48].

In an animal model of cold stress, AntiGan upregulates the immune system, increasing the expression of CD4 and CD28 antigens in T lymphocytes and the phagocytic activity of granulocytes and monocytes [47] (Table 5). 

The results of a pilot clinical trial to study the effect of AntiGan on the levels of tumor markers (TM) in healthy subjects and in subjects with different types of cancer at the time of diagnosis revealed that approximately 50% of the patients presented a reduction in TM levels, and this response was much more evident in patients with cancer, when TM values were above normal levels [46] (Table 5).

Congerin I and II are two proteins isolated from the skin mucus of conger eel (Conger myriaster Brevoort, 1856), which belong to the galectin family of lectin proteins [49,50]. Galectins are a family of carbohydrate-binding proteins defined by their Ca2^+^-independent affinity for β-galactoside sugar. This family of proteins has been suggested to participate in many physiological phenomena such as the development, differentiation, morphogenesis, immunity, apoptosis, and metastasis of malignant cells, and so forth. Congerins from conger eel are components of the biological defense system with agglutinating activity against a marine pathogen, Vibrio anguillarum Bergeman 1909, and with opsonic and cytotoxic activities against cells [51]. The existence of these proteins in the skin mucus of conger eel could be responsible for the immunomodulatory, cytotoxic, and chemopreventive effects obtained in the research studies with AntiGan. The action of other bioactive ingredients in the composition of AntiGan such as polyunsaturated fatty acids [33,34,35] and vitamins, especially vitamin D [36], might support the anti-inflammatory properties of congerins. 

#### 3.1.5. MineraXin (E-MHK-0103)

MineraXin is a proteolipin (PL) derived from the mussel *Mytillus galloprovincialis*, cultivated on the Atlantic coast of Galicia (Spain). The E-MHK-0103 extract is the structural base of MineraXin and is obtained by means of nondenaturing biotechnological procedures that enable all the healthy properties of the original raw material to remain unchanged. MineraXin helps to regulate menopause-related hormones, and to protect against biological decline in women [52].

The nutritional composition of MineraXin includes a high protein content of approximately 64%, is rich in unsaturated fatty acids (69% of the total fatty acids), and approximately 45% corresponds to polyunsaturated fatty acids, it being particularly rich in EPA. The mussel *Mytillus galloprovincialis* Lamarck, 1819 is rich in polar lipid components, constituting 61.5% of the total lipids. The major phospholipid fraction in *M. galloprovincialis* is choline and ethanolamine phosphoglycerides, and ceramide aminoethylphosphonates constitute the third phospholipid fraction in amount [53] (Table 1). Marine omega-3 phospholipids contain n-3 long-chain polyunsaturated fatty acids, differentiating them from phospholipids derived from vegetable sources, since they do not contain long-chain n-3 polyunsaturated fatty acids [54]. Several research papers have described the beneficial effects of marine phospholipids in clinical and pre-clinical studies. Their main effects include an improvement in blood lipids in dyslipidemia, a reduction in arthritic symptoms in osteoarthritis, a reduction in dysmenorrhea in premenstrual syndrome, a reduction in oxidative damage in athletes, and many others that have been described. It seems that marine omega 3 phospholipids are associated with optimal human health and protection against disease [55,56]. 

**Figure 10:**
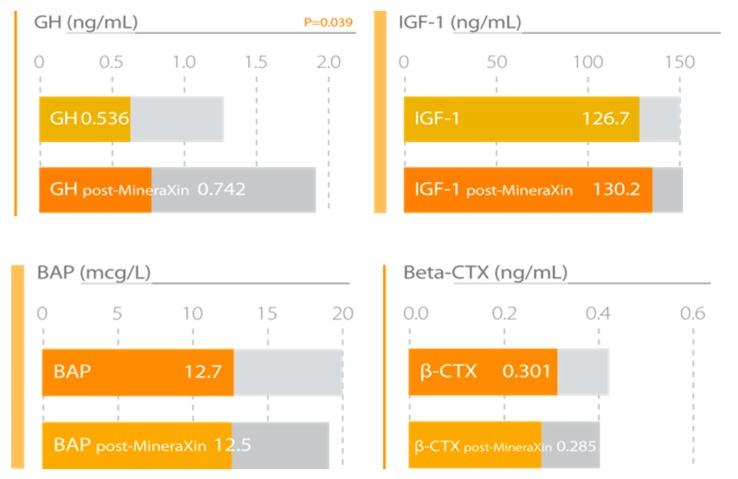
MineraXin enhances bone metabolism, increasing the levels of serum growth hormone (GH) and insulin growth factor-1 (IGF-1) and reducing bone alkaline phosphatase (BAP) and β-crosslaps (β-CTx) concentrations. Study design: three month treatment with 750 mg/day MineraXin in perimenopausal women [52].

**Figure 11:**
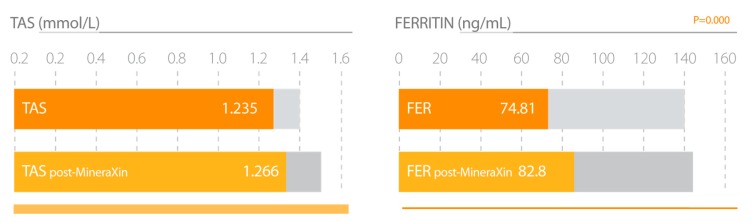
MineraXin increases total antioxidant status (TAS) and iron stores (Ferritin) in perimenopausal women. Study design: three month treatment with 750 mg/day MineraXin in perimenopausal women [52].

Our research studies with MineraXin in menopausal women showed positive effects in general status, frequency of hot flashes, emotional stability, and musculoskeletal pain, possibly associated with its glucosamine-related anti-inflammatory effect [57]. There is clinical evidence of the efficacy of antioxidant nutraceuticals, polyphenols, essential fatty acids, minerals, and cofactors in the treatment of osteoarthritis and other joint-related diseases in humans and animals; many of these antioxidants are natural derivatives of mussels [58]. A significant increase in serum growth hormone (GH) and insulin growth factor-1 (IGF-1) was observed. MineraXin slightly decreases the concentrations of bone alkaline phosphatase (BAP) and calcium and β-crosslaps (β-CTx), indicating a beneficial impact on bone turnover, thereby acting as an antiosteoporotic agent (Figure 10) (Table 6). Recently, a novel osteogenic dodecapeptide peptide (PIE), IEELEEELEAER, was purified from the protein hydrolysate of blue mussels (*Mytilus edulis* Linnaeus, 1758). PIE showed a good reduction in the bone loss in ovariectomized mice, and it also increased the bone mineral density of the ovariectomized mice. PIE accelerates the transformation of cells in the G0/G1 phase into the G2 M phase, which promotes the growth of osteoblasts. PIE (100 μg mL-1) can enhance alkaline phosphatase (ALP) activity by 26.48% compared with the control, and it also inhibits the growth of osteoclasts and tartrate resistant acid phosphatase (TRAP) activity [59]. Furthermore, MineraXin also showed a significant increase in antioxidant status and in ferritin concentration, particularly in those patients with low basal ferritin values (Figure 11) [36]. Chen et al. [60] demonstrated that MCP1-2, a mussel-derived polysaccharide, has good antioxidant activity, supporting our results.

### 3.2. Plant Biotechnology-Related Products

#### AtreMorine (E-PodoFavalin-15999)

AtreMorine is a novel compound, obtained by means of non-denaturing biotechnological procedures from structural components of the *Vicia faba* L. plant. AtreMorine is a powerful enhancer of plasma catecholamines (noradrenaline, adrenaline, dopamine), with no apparent effect on serotonin. The E-PodoFavalin-15999 extract is the structural base of Atremorine and is a natural source of L-DOPA; it also contains a plethora of other bioactive substances such as vegetal proteins, unsaturated fatty acids, minerals, and vitamins, vegetal fiber, starch, vegetal pigments (carotenes), and vegetal sterols (phytosterols) (Table 1). 

In vitro studies revealed that AtreMorine is a powerful neuroprotectant in (i) human neuroblastoma SH-SY5Y cell cultures; (ii) hippocampal slices under conditions of oxygen and glucose deprivation; and (iii) striatal slices under conditions of 6-OHDA-induced neurotoxicity [61]. AtreMorine displayed neuroprotective and anti-inflammatory effects in different in vitro models, suggesting that this novel bioproduct may be of use in the protection of neurons against neurodegenerative processes, especially dopaminergic neurons associated with Parkinson´s disease (PD) pathogenesis (Table 7).

**Figure 12:**
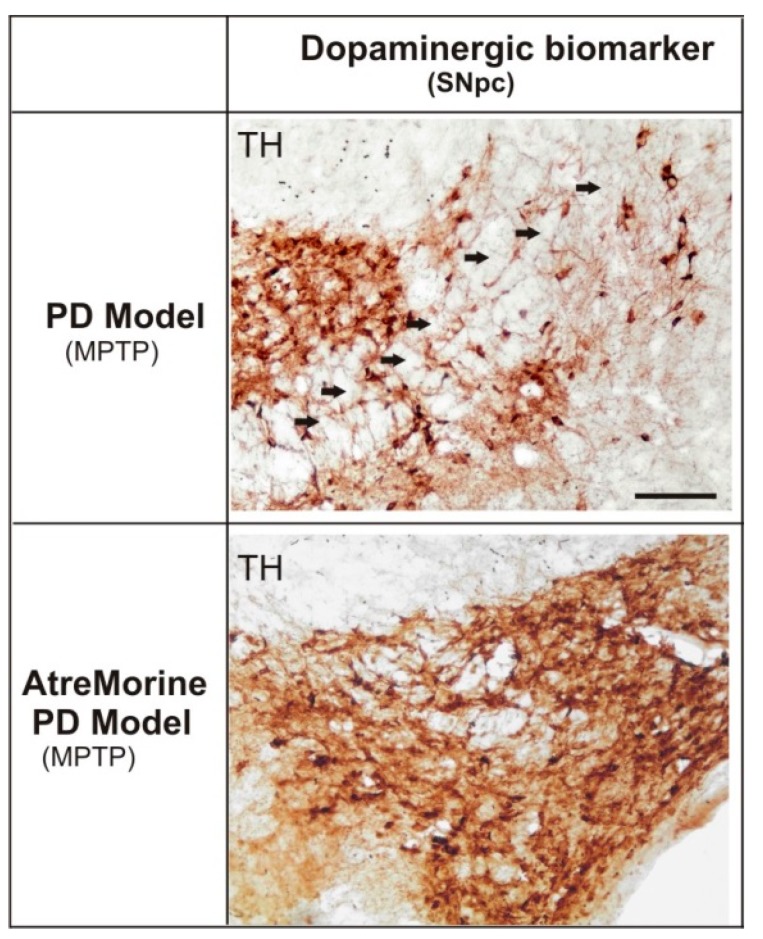
Effect of AtreMorine treatment in 1-methyl-4-phenyl-1,2,3,6-tetrahydropyridine (MPTP)-induced mouse model of Parkinson’s disease. Comparative photomicrographs of dopaminergic immunoreactivity (TH) of the substantia nigra pars compacta (SNpc) of MPTP-induced mice with and without AtreMorine treatment. Transverse brain sections of mice from both groups (control) and (AtreMorine), showing the remarkable neuroprotective effect of AtreMorine treatment by reducing the dopaminergic degeneration in the SNpc neurons. Scale bar: 100 µm. Adapted from [62].

*In vivo* studies showed that AtreMorine (i) protects against 1-methyl-4-phenyl-1,2,3,6-tetrahydropyridine (MPTP)-induced dopaminergic neurodegeneration; (ii) inhibits MPTP-induced microglia activation and neurotoxicity in the *substantia nigra*; and (iii) improves motor function in mice with MPTP-induced neurodegeneration (Figure 12) [62] (Table 7).

**Figure 13:**
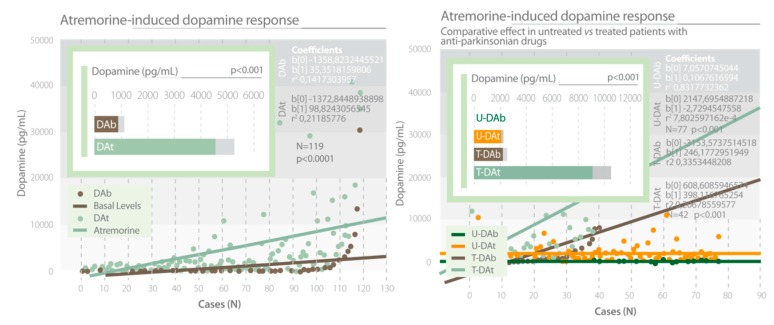
AtreMorine increases the levels of dopamine in Parkinson’s disease. Study in both untreated patients and patients chronically treated with conventional antiparkinsonian drugs. DAb: Basal dopamine levels; DAt: Plasma dopamine levels at one hour after AtreMorine administration (5 g, p.o.) [64]. U-DAb: Basal dopamine levels in patients never treated before with antiparkinsonian drugs. U-DAt: Plasma dopamine levels in untreated patients one hour after AtreMorine administration (5 g, p.o.). T-DAb: Basal dopamine levels in patients chronically treated with antiparkinsonian drugs. T-DAt: Plasma dopamine levels in patients chronically treated with antiparkinsonian drugs one hour after AtreMorine administration (5 g, p.o.) [63].

Clinical studies showed that AtreMorine is an enhancer of dopaminergic neurotransmission, increasing plasma dopamine levels by 200- to 500-fold in patients with PD and related movement disorders. This effect is observed in untreated patients who receive AtreMorine for the first time (never treated before with antiparkinsonian drugs) and in patients chronically treated with L-DOPA or other antiparkinsonian drugs (Figure 13) [14,63,64]. AtreMorine is an option for the reduction and minimization of the potential side effects of conventional antiparkinsonian drugs such as the “wearing-off” phenomenon, motor fluctuations and dyskinesia, systemic complications (gastrointestinal disorders, cardiovascular problems, hormonal dysregulation), and neuropsychiatric disorders (depression, anxiety, toxic psychosis). The co-administration of AtreMorine with other antiparkinsonian drugs enables a dose reduction of the conventional drugs by 25–50%, with an enhancement of the clinical benefits, a reduction of short- and long-term adverse drug reactions, and it also extends the therapeutic effect of conventional antiparkinsonian drugs. A recent study reported that AtreMorine-induced dopamine response is pharmacogenotype-specific and lasts from six to 12 hours, depending upon the pharmacogenetic profile of each patient (environmental factors, nutrition, and co-administration of different drugs may also modify this response). Genetic variants in pathogenic genes (*APOE*, *LRRK2*), metabolic genes (*CYP2D6*, *CYP2C9*, *CYP3A5*, *NAT2*) and detoxification genes (*CYP1B1*, *GSTP1*, *SOD2*) influence the response of dopamine to AtreMorine [65] (Table 7).

Recent attempts have been made using novel compounds (alternative and complementary medicines) for the treatment of PD [66]. The revival of some classic and novel natural products (i.e., *Vicia faba* L., *Mucuna pruriens* L., *Glycyrrhiza glabra* L.*, Uncaria rhyncophylla* Miq.*, Siegesbeckia pubescens* Makino, *Carthamus tinctorius* L., *Curcuma longa* L., *Centella asiatica* L., flavonoids, ginsenosides, cyanidin-3-O-glucoside, caffeine, resveratrol, and other polyphenols) has also been proposed; new applications have been submitted to the USA and European Patent Offices in this regard. Some of these products show selective dopaminergic neuroprotection, with additional benefits such as antioxidant, anti-inflammatory, and neurotrophic effects. A recent study demonstrated the inhibitory effects induced by *Vicia faba* extract on oxidative stress biomarkers and dopamine turnover in the striatum, which is likely related to its phenolic content [67]. A previous study using a mammalian neuronal cell line showed the potent antioxidant and neuroprotective roles of phytosterols [68]. Vitamins such as beta-carotenes or vitamin B6 also have antioxidant properties, and evidence from two case-control studies shows that they decrease the risk for PD [69,70]. Unsaturated fatty acids and other plant-derived micronutrients reduce proinflammatory cytokine production and may reduce inflammation in these patients [71]. In conclusion, the pro-dopaminergic effect of AtreMorine can be attributed to the rich content of natural L-DOPA (average concentration 20 mg/g) in its composition. However, the neuroprotective effect of this nutraceutical product on dopaminergic neurons, as demonstrated in in vitro studies and in animal models of PD, cannot be attributed to L-DOPA alone, but to other intrinsic constituents (selective neurotrophic factors) of the compound [63].

## 4. Discussion

The presence of functional components in food used in the prevention, delay, or amelioration of diseases, or in complementing other pharmacological treatments under medical supervision, is one of the most important fields in nutrition research from a human health perspective. Nutritional status plays a key role in relation to important physiological processes such as mucosal integrity and barrier function, cognitive function, and immune response as well as in immune disorders, chronic inflammation, frailty, sarcopenia and aging, and cognitive decline. Nutritional status can also affect resilience, susceptibility, and response to therapy. Consequently, a poor nutritional status, caused either by an unhealthy diet or by malabsorption of nutrients, is a major risk factor for many chronic diseases.

The development of nutraceuticals from marine and plant species under non-denaturing conditions and maintaining their original food matrix for their further transformation into a pharmaceutical form are the main characteristics of the nutraceuticals we have developed. Non-denaturation conditions and the use of the original matrix of the species allow all health-beneficial properties to remain intact.

Fish occupies the highest position in marine animal consumption and is important to the global economy. In 2012, fish provided approximately 16% of the world’s protein requirements with herring, salmon, cod, flounder, tuna, mullet, and anchovy being the most common species of fish used for food [72]. Many researchers found strong links between fish and seafood consumption and positive health effects, particularly in a decreased risk of coronary heart and cardiovascular diseases, a decrease in inflammatory diseases such as arthritis, and the prevention of cancer [73,74,75]. Historically, the main benefits of fish consumption were attributed to its high long-chain n-3 PUFA content, but research is yielding ever more proof that other nutrients from fish also have positive effects on human health. Fish protein has been considered to have a high nutritional value, is highly digestible, and rich in several peptides and essential amino acids that are limited in meat proteins such as methionine and lysine [76]. Fish is also rich in minerals such as calcium, iron, selenium, phosphorus, and magnesium, and vitamins such as A, D, B_3_, B_6_, B_12_, B_2_, and E [77]. According to the Food and Agriculture Organization (FAO)/World Health Organization (WHO) Joint Expert Consultation, there is convincing evidence that fish consumption (i) reduces the risk of death from coronary heart disease; (ii) in women, reduces the risk of sub-optimal neurodevelopment in their offspring; (iii) reduces the risk of other adverse health outcomes including ischemic stroke, non-fatal coronary heart disease events, congestive heart failure, atrial fibrillation, cognitive decline, depression, anxiety, and inflammatory diseases; and (iv) provides a greater nutritional impact than the sum of the health benefits of the individual nutrients consumed separately [78]. Mollusks are also widely consumed as marine foods and are considered natural functional foods. Lipidic and aminoacidic extracts derived from mussels have been related to anti-inflammatory properties in different pathologies, observing beneficial effects on hypertension, osteoarthritis, skin burns, asthma, or wound healing [52,79,80,81,82,83]. Fish, mollusks, and crustaceans are amongst the richest sources of bioactive molecules [84]. In this regard, bioactive lipid mediators, recently termed resolvins, maresins, and protectins, are a group of molecules derived from the omega-3 fatty acids DHA and EPA, with both anti-inflammatory and proresolving activity in acute or chronic inflammatory-related diseases [85]. 

In general, when considering human nutrition and health-related aspects, it is important to consider that foods are formed by combinations of nutrients and other bioactive molecules that work together, and influence our health in complex and highly interactive ways. Most probably, the effects of fish on human health discussed herein are due to the consumption of the fish as a whole, and hence to the combination of all the nutrients and bioactive compounds present in the foodstuff [76,86,87]. The consumption of complex marine extracts provides numerous health benefits concerning the immune system, hypertension, atherosclerosis, arthritis, and menopause-related dysfunctions, and might reduce the risk of cardiovascular problems, osteoporosis, or senile muscle weakness, as we observed in our research studies. 

Among the vegetal extracts, E-PodoFavalin-15999 (AtreMorine) represents a natural approach to the treatment of Parkinson´s disease and hypo-dopaminergic-related disorders. AtreMorine contains natural L-DOPA and is a good complement to conventional treatments in PD. This naturally-occurring bioactive extract caused a rapid increase in plasma dopamine levels in PD patients, and in animal models it is neuroprotective and anti-inflammatory.

The mechanism of action of these extracts has not been fully explored, and further research is necessary to completely understand the effects of the different bioactive nutrients and compounds as well as the synergistic effects from the combined intake of them all. An ‘extract’ contains major active constituents of the original source, in a highly concentrated way. The amount of the compound may be thousands of times higher than that originally found in the vegetable or fish. This feature enables the dose of a given compound (nutraceutical or supplement) to be increased in order to make it really effective, to provide a healthy benefit, and to minimize the presence of certain diseases. Otherwise, the organism would have to consume substantial quantities of certain foods in order to achieve the same goal. 

Worldwide acceptance of these facts supports the rise of the nutraceuticals market. The global market of the nutraceutical industry, according to Mordor Intelligence, was valued at around 205.39 billion USD in 2016 and is expected to reach around USD 294.79 billion in 2022, with a compounded annual growth rate (CAGR) of 6.3% between 2017 and 2022 [88,89]. The North American market is the largest market in the global nutraceutical industry, with a CAGR of 7.1% expected during the 2018–2023 target period. The European nutraceutical market is expected to register a CAGR of 7.5%, during the forecast period (2019–2024). Germany holds the maximum market share of 14%, followed by the United Kingdom and France [90]. In the European region, nutraceuticals are gaining importance and becoming part of the daily consumer diet. The reason for this change in dietary behavior is due to the increased prevalence of lifestyle-related diseases and the role of nutrition in the prevention of risk factors associated with the development of these diseases. 

The most researched and most interesting aspects of nutraceuticals are their use in medicine, for the prevention of, or to support the treatment of a variety of diseases such as cancer, osteoarthritis, cardiovascular disorders, etc. The use of nutraceuticals and functional foods in prevention could lead to a decrease in drug dependence, associated with multiple adverse reactions. Both can be included in the usual diet of any person, but they are especially indicated in populations with special nutritional needs (pregnant women and children), deficiencies, certain food intolerances, risk of several diseases (cardiovascular, gastrointestinal, osteoporosis, diabetes, etc.), and elderly people. Health professionals and nutritionists should strategically work together to plan appropriate regulations to provide the ultimate health and therapeutic benefits for mankind. 

The interaction of food, health, and genetics is an area that should be taken into consideration. Based on nutrigenomic and epigenomic principles, nutritional advice can benefit the relationship between genes and the nutritional habit-related consequences [91,92,93]. Nutritional genomics studies these interactions through three sub-branches: *Nutrigenetics*: studying how our genes and genetic variation can influence nutritional needs, in other words, the amount of nutrients required by each individual for good health; *Nutrigenomics*: studying how the food we eat (and the nutrients that we absorb) can affect genes and how genes are expressed in the form of individual characteristics (phenotype); and *Epigenetics*: studying how external factors such as diet, physical activity, environmental pollution or toxins, etc. may modify the expression of our genes (phenotype), without changing our DNA. Experts on these topics are investigating the potential of personalizing diet to improve our health and to create diets based on individual genetic profiles. Medical or nutrition professionals can also use this scientific knowledge to personalize functional food and nutraceuticals, aiming to achieve more effective natural therapies [94]. In previous studies, we observed the different behavior or efficiency of nutraceuticals according to the genomic profile of the patients. A differential pattern of cholesterol response to DefenVid is associated with the *IL1B-T3954C*, *IL6-G174C*, *IL6R-A1510C*, and *TNFA-G308A* variants, which are involved in inflammatory reactions associated with atherogenesis. The *APOE*-dependent antiatherogenic effect of LipoEsar supports the application of genetic studies to improve the response to nutraceuticals. The patients´ response to Atremorine was also modulated by the pharmacogenetic profile of each patient: CYP2D6-Poor metabolizers exhibit the lowest basal dopamine levels and response to Atremorine while CYP2D6-Ultra Rapid metabolizers show the highest basal dopamine levels and the most spectacular response to Atremorine; however, in the case of CYP2C9 and CYP3A4/5 enzymes, CYP2C9-Intermediate metabolizers and CYP3A4/5-Intermediate metabolizers are the best responders and CYP2C9-Poor metabolizers and CYP3A4/5-Rapid metabolizers are the worst responders to Atremorine.

## 5. Conclusions

Academic, governmental, and private research institutes around the world are devoting substantial efforts to identifying how functional foods and food ingredients can help prevent chronic diseases or optimize health, which would reduce health care costs and improve the quality of life of many consumers. In recent years, the concept of personalized nutrition has gained special relevance in the field of health. Identifying and promoting optimal diets and lifestyles for people, taking into account their genetic traits, should be essential for the prevention and treatment of chronic diseases [95]. Although a higher level of evidence will be required in the coming years, nutrigenomics will increase this level of evidence and personalized nutrition will be possible and successful. The development of nutraceuticals for the prevention and management of diseases in individuals with different genetic profiles is discussed in the context of the opportunities that occur in the nutrigenetic/pharmacogenetic interface, leading to personalized nutrition [96]. The use of each person’s genetic profile for the prescription of nutraceuticals is a major goal to be striven for in order to improve their effectiveness in the prevention and natural treatment of prevalent diseases. 

## Figures and Tables

**Table 1:** Nutritional properties of nutraceuticals.

	MARINE LINE					VEGETABLE LINE
NUTRACEUTICALS	LipoEsar (E-SAR-94010)	DefenVid (E-JUR-94013)	CabyMar (E-CAB-94011)	AntiGan (E-Congerine-10423)	MineraXin (E-MHK-0103)	Atremorine (E-PodoFavalin-15999)
**Origin**	*Sardina pilchardus* (Dorsal muscle)	*Trachurus trachurus* (Dorsal muscle)	*Scomber scombrus* (Dorsal muscle)	*Conger conger* (Skin and muscular structures)	*Mytillus galloprovincialis*	*Vicia faba* L. (Structural components)
**Bioactive properties**	**Atheroprotector** **Immune-enhancing properties**	**Immunorregulator** **Anti-inflammatory**	**Bioenergizing Antioxidant** **Anti-inflammatory**	**Apoptotic** **Carcinogenic**	**Antiosteoporotic** **Antioxidant** **Hormonal regulator**	**Pro-dopaminergic** **Antioxidant** **Neuroprotector**
Nutritional composition	**Protein** 65% **Unsaturated FA** (% of total) 11% EPA, DHA, Oleic **Saturated FA** (% of total) 9% **Vitamins** C, D, B complex, inositol **Minerals** Potassium Phosphorous Calcium Sodium Magnesium Zinc Iron Manganese Copper	**Protein** 70% **Unsaturated FA** (% of total) 12% EPA, DHA, alpha-Linolenic, Oleic **Saturated FA** (% of total) 7% **Vitamins** A, C, D, B complex, inositol **Minerals** Calcium Potassium Phosphorus Magnesium Iron Iodine Zinc Copper	**Protein** 65% **Unsaturated FA** (% of total) 18% **Monounsaturated FA** (% of fat) **LCMUFA 58%** cis-gondoic acid cis-cetoleic acid **Polyunsaturated FA** (% of fat) 21% EPA, DHA, Linoleic **Saturated FA** (% of total) 6% **Vitamins** B complex (B_5_, B_6_, B_12_), C, D, E **Minerals** Phosphorus Sodium Magnesium Calcium Copper Iron Potassium Manganese Zinc	**Protein** 80% **Unsaturated FA** (% of fat) 70% **Monounsaturated FA** Oleic, palmitoleic, gadoleic **Polyunsaturated FA** DHA, Linoleic EPA **Saturated FA** (% of fat) 30% **Vitamins** A, D, B complex (B_1_, B_2_, B_3_) **Minerals** Phosphorus Calcium Potassium Magnesium Iron Zinc	**Protein** 65% **Unsaturated FA** (% of fat) 69% mostly EPA **Polyunsaturated FA** (% of fat) 45.4% **Monounsaturated FA** (% of fat) 23.6% **Saturated FA** (% of fat) 31% **Phospholipid fraction** 61.5% choline, ethanolamine phosphoglycerides and ceramide aminoethylphosphonates **Vitamins** B complex (B_2_, B_5_, B_9_, B_12_) **Minerals** Phosphorus Calcium Potassium Sodium Magnesium Iron Zinc Manganese Selenium Copper	**Protein** 65% **L-Dopa** 20 mg/gr extract **Carbohydrates** 66% **Dietary fiber** 30% **Monounsaturated FA** 29% (% of fat) **Polyunsaturated FA** 41.1% (% of fat) **Saturated FA** (% of fat) 29.8% **Pigments Carotenoids** Lutein, β-Carotenes, Epoxides, t-Zeaxanthin **Phytosterols** β-sitosterol, Campesterol Stigmasterol, Sitostanol, Cholesterol **Vitamins** B complex (B_2_, B_3_, B_5_, B_6_, B_9_), C, E, K **Minerals** Manganese Potassium Calcium Sodium Magnesium Iron Zinc Copper Selenium

**Table 2:** LipoEsar: Summary of basic and clinical studies.

LipoEsar		
Study	Characteristics	Results–Conclusions
Lombardi, V.R.M. et al., 1999 [13].	Sixty male Sprague-Dawley rats. Three groups of rats. One with normal diet, and two groups supplemented with E-SAR-94010, 1 mg and 5 mg. Experimental time 12 weeks. Biochemical study. Experimental group 10 healthy volunteers. Two sequential doses of E-SAR-94010 during two weeks each (1500 mg/day and 500 mg/day in soft gelatinous capsules). Biochemical and immunological analyses at baseline and at weeks 1 and 2. Participants continued their usual diet.	1. No changes in rats body weight. 2. Significant changes in cholesterol and triglycerides levels comparing to rats control group at weeks 8 and 12. 3. In healthy volunteers, significant differences in HDL and LDL levels were observed at the end of the study between the group of 500 mg and 1500 mg. 4. The lymphocyte markers showed a significant increase in CD3, CD4 y CD25 antigen expression in both treatment groups.
Lombardi, V.R.M. et al., 2001 [18].	Fourteen Sprague-Dawley rats. Two groups of 7 rats. Group 1 with standard diet. Group 2 supplemented with 50 mg/day of E-SAR-94010. Experimental time 12 weeks (6 weeks before pregnancy and 6 weeks after pregnancy). Intragastric treatment administration. Biochemical and immunological studies.	1. E-SAR-94010 significantly reduced triglycerides levels in the pups and their mothers of the supplemented group with respect to control group. 2. No significant differences in the atherogenic index between both experimental groups. 3. E-SAR-94010 increased levels of CD25, CD28, CD54 and CD56 on lymphocytes from the supplemented group of rats and their pups.
Cacabelos, R. et al., 2004 [19].	Description of the principal clinical effects of LipoEsar. Duration of treatment 1–3 months Daily dose of 250–500 mg. APOE genotype dependent effects of LipoEsar in 419 dyslipemic patients.	1. LipoEsar reduced blood total cholesterol, glucose, uric acid, triglycerides, ALT and AST. 2. LipoEsar diminished the size of xanthelasma plaques after 6–9 months of treatment. 3. The therapeutic response of patients with dyslipemia to LipoEsar is APOE-related.

**Table 3:** DefenVid: Summary of basic and clinical studies.

DefenVid		
Study	Characteristics	Results–Conclusions
Lombardi, V.R. et al., 2002 [30].	Experimental group 300 pigs. Fifteen groups of 20 pigs each. Five dietary treatments with different amount of fish extracts, sardine, mackerel and E-JUR-94013 (horse mackerel), in their habitual diet. Duration of the study 42 days. Anthropometric, biochemical and immunological analysis.	1. E-JUR-94013 regulated IgA synthesis or release. 2. E-JUR-94013 reduced cholesterol levels in the sera of treated pigs. 3. E-JUR-94013 produced significant differences in feed intake and feed:gain ratio with respect to control group. 4. E-JUR-94013 increased the levels of IgA, IgM and IgE with respect to control group.
Lombardi, V.R. et al., 2005 [23].	Experimental group 24 healthy volunteers. Evaluation of the effect of E-JUR-94013 on in vitro peripheral blood lymphocytes activation, FAS expression and apoptosis.	1. E-JUR significantly increased the levels of CD25, CD8, CD38, CD19 and HLA-DR antigen expression. 2. E-JUR significantly reduced the percentages of apoptotic CD19 CD38 double positive lymphocytes.
Lombardi, V.R.M et al., 2018 [31].	Three clinical studies to evaluate the effect of E-JUR-94013 in the improvement of immune system function. Study #1 50 patients. Study #2 205 patients. Study #3 1500 patients. Treatment regimen 750 mg/day of E-JUR-94013. Duration of treatment: Study #1 6 months; Study #2 and #3 3 months. In Study#2 patients were divided in 2 subgroups according to the number of leukocytes, high or low relative to reference ranges. In Study #3 a genetic analysis is incorporated to investigate the effect of genes in the response to DefenVid.	1. Study #1 showed an increase in all leukocyte subclasses with a significant increase in the number of neutrophils and eosinophils. Serum immunoglobulins A, G and M were increased. A slight reduction in the concentration of IgE was found. 2. Study #2 showed an immunomodulatory effect. with an increase in white cells in the immunodeficient group and a decrease in white cells in the group with high leukocyte count at baseline, bringing the white cell count to normal ranges in both cases. 3. In Study #3 DefenVid was affected by polymorphic variations of genes involved in immune regulation of inflammation.

**Table 4:** CabyMar: Summary of basic and clinical studies.

CabyMar		
Study	Characteristics	Results - Conclusions
Lombardi, V.R. et al., 2005 [23].	Experimental group 24 healthy volunteers. Evaluation of the effect of E-CAB-94011 on in vitro peripheral blood lymphocytes (PBL) activation, FAS expression and apoptosis.	1. E-CAB significantly increased the levels of CD25, CD8, CD38, CD19 and HLA-DR antigen expression. 2. E-CAB significantly reduced the percentages of apoptotic CD19 CD38 double positive lymphocytes.
Lombardi, V.R. et al., 2005 [37].	Experimental group 360 piglets divided in 4 groups (Group 1 normal diet, Group 2 supplemented diet with 0,1 g/Kg E-CAB, Group 3 supplemented with 0,25 g/Kg E-CAB, and Group 4 supplemented with 0,5 g/Kg). Anthropometric, biochemical and immunological analysis. Duration of the study 56 days.	1. E-CAB-94011 increased total body weight. 2. E-CAB-94011 induced significant changes in total cholesterol, LDL, and hepatic transaminases. 3. E-CAB-94011 increases A and G immunoglobulin levels. 4. E-CAB-94011 increased CD4, CD8, CD25 and CD56 lymphocyte antigen expression.
Cacabelos, R. et al., 2018 [16].	Mice treated with CabyMar. Biochemical, histological, and gene expression analysis.	1. CabyMar significantly increased urea and creatinine levels and decreased glucose and lactate levels. 2. CabyMar significantly increased gene expression biomarkers in muscle and heart tissue.

**Table 5:** AntiGan: Summary of basic and clinical studies.

AntiGan		
Study	Characteristics	Results - Conclusions
Lombardi, V.R.M. et al., 2006 [47].	Six Sprague-Dawley rats. Acute experimental model of immune system stress induced by exposure of rats to a hypothermic shock. Whole blood from rats was studied under two different concentrations of AntiGan (20 and 100 µg/mL) during 4, 16 and 24 hours. Analysis of T lymphocytes markers, and phagocytic activity.	1. Increased CD4 and CD28 antigen expression in both groups of treatment. 2. Increased percentage of CD4 CD25 double positive T lymphocytes. 3. Increased phagocytic activity (monocytes and granulocytes) in both groups of treatment.
Lombardi, V.R.M. et al., 2018 [45].	Evaluation of the apoptogenic activity of AntiGan after 24 h of incubation (10, 25 and 50 μL/mL AntiGan) of HL-60, Hs 313.T, SW-480, Caco-2 and HT-29 cell lines using growth inhibition and apoptosis activity assays. In vivo studies in mice (n = 56) by inducing colitis with oral administration of 2% dextran sulphate sodium (DSS) for 6 weeks. AntiGan was administered integrated in the diet as pellet biscuits (2,5%, 5%, 10% AntiGan).	1. AntiGan treatment inhibited growth in tumor cell lines. 2. AntiGan induced apoptosis in tumor cell lines. 3. AntiGan downregulated Bcl-2 gene expression. 4. AntiGan displayed a powerful anti-inflammatory effect in DSS-induced colitis.
Lombardi, V.R.M. et al., 2019 [46].	The effect of E-Congerine-10423 on tumor markers was studied in healthy subjects (n = 50) and in patients with different types of cancer (n = 156) at the time of diagnosis. AntiGan treatment 750 mg/day.	1. AntiGan produced, in about 50% of the patients, a reduction in the levels of tumor markers, especially in patients with cancer.

**Table 6:** MineraXin: Summary of basic and clinical studies.

MineraXin		
Study	Characteristics	Results–Conclusions
Corzo, L. et al., 2017 [52].	Experimental group 91 perimenopausal and postmenopausal women. MineraXin treatment left 750 mg/day during 3 months. Analysis of clinical symptoms, hormonal status, hypothalamic-pituitary-bone axis, markers of bone formation and resorption, antioxidant status, iron stores, cortisol, and BMI.	1. Significant improvement in general status, hot flash frequency, emotional stability and musculoskeletal pain. 2. Increased serum estradiol and inhibin-A concentrations. 3. Decreased FSH and LH levels. 4. Increased serum GH and IGF-1 levels. 5. Slight increase in bone formation and a moderate decrease in bone resorption. 6. Significant increase of antioxidant status. 7. Increased ferritin concentrations.

**Table 7:** AtreMorine: Summary of basic and clinical studies.

AtreMorine		
Study	Characteristics	Results–Conclusions
Cacabelos, R. et al., 2016 [62].	Experimental group 119 patients with parkinsonian disorders. Two groups of patients, untreated *vs*. chronically treated patients. Oral dose of AtreMorine 5 g. Analysis of dopamine levels. APOE and CYP genetic studies.	1. A single oral dose of AtreMorine (5g) induced a significant increase in dopamine levels after 1 hour. 2. The AtreMorine-induced dopamine response was different in APOE and CYP genetic variants.
Cacabelos, R. et al., 2016 [63].	Experimental group, 119 patients with parkinsonian disorders. Two groups of patients, untreated *vs*. chronically treated patients. Oral dose of AtreMorine, 5 g. Analysis of neurotransmitters and hormones levels.	1. A single oral dose of AtreMorine (5g) induced a significant increase in dopamine, noradrenalin and adrenaline levels after 1 hour. 2. AtreMorine induced a significant decrease in the levels of prolactin, GH, and cortisol.
Carrera, I. et al., 2017 [64].	Experimental group, wild type C57BL6/J mice. MPTP-induced mice model of Parkinson Disease. Duration protocol 7 weeks. AtreMorine at doses of 2 mg and 4 mg was administered integrated in pellet biscuits. Immunohistochemical analysis. Motor function analysis.	1. AtreMorine protected against MPTP-induced dopaminergic Neurodegeneration. 2. AtreMorine inhibited microglial activation and cell death in the *Substantia Nigra*. 3. AtreMorine improved motor and cognitive functions in MPTP mice model.
Cacabelos, R. et al., 2019 [65].	Experimental group, 183 patients with parkinsonian disorders. Oral dose of AtreMorine, 5 g. Study of the influence of genes involved in pathogenic, metabolic, transporter, pleiotropic, and detoxifying functions.	Genetic variants in pathogenic genes (*APOE*, *LRRK2*), metabolic genes (*CYP* Family and *NAT2*) and detoxification genes (*CYP1B1*, *GSTP1*, and *SOD2*) influence the response of dopamine to AtreMorine.
Romero, A. et al., 2017 [61].	Evaluation the neuroprotective and antiinflammatory effects of AtreMorine in in vitro models of Parkinson Disease and oxidative stress. 6- OHDA Parkinson model and rote/oligo, oxidative stress model in Neuroblastoma SH- SY5Y cells. 6- OHDA Parkinson model in rat striatal slices. OGD/Reox as an oxidative stress model in rat hippocampal slices. LPS as a model of neuroinflammation in BV2 microglia cells.	Atremorine showed neuroprotective and antiinflammatory effects in different in vitro models.
